# CT-Based Radiomics Signatures for Predicting the Risk Categorization of Thymic Epithelial Tumors

**DOI:** 10.3389/fonc.2021.628534

**Published:** 2021-02-26

**Authors:** Jin Liu, Ping Yin, Sicong Wang, Tao Liu, Chao Sun, Nan Hong

**Affiliations:** ^1^ Department of Radiology, Peking University People’s Hospital, Beijing, China; ^2^ Pharmaceutical Diagnostic Team, GE Healthcare, Shanghai, China

**Keywords:** radiomics, thymic epithelial tumors, pathologic classification, computed tomography, machine learning

## Abstract

**Objectives:**

This study aims to assess the performance of radiomics approaches based on 3D computed tomography (CT), clinical and semantic features in predicting the pathological classification of thymic epithelial tumors (TETs).

**Methods:**

A total of 190 patients who underwent surgical resection and had pathologically confirmed TETs were enrolled in this retrospective study. All patients underwent non-contrast-enhanced CT (NECT) scans and contrast-enhanced CT (CECT) scans before treatment. A total of 396 hand-crafted radiomics features of each patient were extracted from the volume of interest in NECT and CECT images. We compared three clinical features and six semantic features (observed radiological traits) between patients with TETs. Two triple-classification radiomics models (RMs), two corresponding clinical RMs, and two corresponding clinical-semantic RMs were built to identify the types of the TETs. The area under the receiver operating characteristic curve (AUC) and accuracy (ACC) were useful to evaluate the different models.

**Results:**

Of the 190 patients, 83 had low-risk thymoma, 58 had high-risk thymoma, and 49 had thymic carcinoma. Clinical features (Age) and semantic features (mediastinal fat infiltration, mediastinal lymph node enlargement, and pleural effusion) were significantly different among the groups(*P* < 0.001). In the validation set, the NECT-based clinical RM (AUC = 0.770 for low-risk thymoma, 0.689 for high-risk thymoma, and 0.783 for thymic carcinoma; ACC = 0.569) performed better than the CECT-based clinical-semantic RM (AUC = 0.785 for low-risk thymoma, 0.576 for high-risk thymoma, and 0.774 for thymic carcinoma; ACC = 0.483).

**Conclusions:**

NECT-based and CECT-based RMs may provide a non-invasive method to distinguish low-risk thymoma, high-risk thymoma, and thymic carcinoma, and NECT-based RMs performed better.

**Advances in Knowledge:**

Radiomics models may be used for the preoperative prediction of the pathological classification of TETs.

## Introduction

Thymic epithelial tumors (TETs) originate from thymic epithelial cells and are the most common tumors in the anterior mediastinum, accounting for 47% of mediastinal neoplasms (1). The most common age of patients is 35–70 years, with no significant difference in sex. Approximately one-third of patients have myasthenia gravis (2).

The World Health Organization (WHO) histological classification of TETs is complex. Before 1980, TET classification was based on the morphological features of tumor cells and was divided into spindle cell, lymphocyte dominant, epithelial cell, and lymphoepithelial types. In 1999, the WHO revised the classification by tissue origin and function and adopted the classification proposed by the German pathologist Muller–Hermelink. Then, TETs were divided into types A, AB, B1, B2, and B3 and thymic cancer. Based on the consensus of the international thymic malignancy interest group on thymic tumors, the 2015 WHO classification of TETs modified the view of thymoma as a benign tumor, except for nodule-type thymoma and micro thymoma with lymphoid stroma. Moreover, other thymomas are considered malignant tumors (3). Therefore, the main treatment for TETs is surgical excision. Previous studies have found that type B2 and B3 thymomas are less likely to be completely removed than type A, AB, or B1 thymomas because of their more aggressive behavior (4). Moreover, patients with types B2 and B3 thymomas had higher tumor recurrence rates and mortality rates than those with other types (5). According to clinical needs, Jeong et al. (6) simplified the TET classification into low-risk thymoma (A, AB, and B1), high-risk thymoma (B2 and B3), and thymic cancer. Recently, some scholars found that there was a significant correlation between the new tumor nodes metastasis (TNM) staging system and the WHO histological grade (7). The potential for complete resection and the overall and disease-free survival outcomes were closely related to the thymoma stage. Furthermore, both the histotype and stage correlated with disease-free survival. Therefore, accurate preoperative classification can help develop individualized treatment methods for TET patients and improve prognosis (8, 9).

CT is the most important imaging method in the diagnosis of TETs. The CT features of non-invasive TETS are as follows: round or oval mass, usually located on one side of the anterior superior mediastinum; intact capsule, uniform density, clear surrounding fat space; and homogeneous light to moderate enhancement on the enhanced scan. The CT signs of invasive TETS are as follows: irregular mass with unclear margin; an incomplete capsule, peritumoral fat deposition; visible calcification; obvious uneven enhancement on enhanced scans; pleural effusion; pericardial effusion; and displacement and compression of cardiac vessels (9). The differential diagnosis includes mainly the following: 1) anterior mediastinal lymphoma, mediastinal lymphoma with the nodular fusion of multiple lymph nodes, the uneven density of lesions, rare calcification, swelling of adjacent lymph nodes and displacement of adjacent vessels; showing mild to moderate enhancement on enhanced scans; and 2) teratoma, mostly located in the middle of the anterior mediastinum and containing fat, bone, calcification, and soft tissue components; showing heterogeneous enhancement.

Radiomics models (RMs) have been widely used to predict tumor type and stage, lymph node metastasis, and prognosis (10–19), specifically in lung, breast, and colorectal cancer. Previous studies have shown that texture analysis based on CT images can distinguish high-risk thymoma from low-risk thymoma (20). However, thymic cancer has not been included. Because identifying thymic cancer *via* conventional imaging is difficult and accurate preoperative identification facilitates the development of individualized treatment approaches, a simple non-invasive method of identification would be of great clinical benefit.

Different high-dimensional quantitative radiomics features can be combined into predictive RMs to quantify tumor heterogeneity and show underlying malignant features (21, 22). Moreover, previous studies have shown that low-risk thymoma, high-risk thymoma, and thymic carcinoma are associated with variations in the morphology of epithelial cells, the ratio of lymphocytes to epithelial cells, invasiveness, and gene expression (3, 23, 24). Overexpression of specific genes is common in thymic carcinoma but rare in thymoma, and the expression levels of these genes are related to the degree of malignancy, biological characteristics, and prognosis of the patient. Therefore, we hypothesized that CT-based radiomics signatures can distinguish among low-risk thymoma, high-risk thymoma, and thymoma. The purpose of this study was to assess the performance of radiomics approaches based on CT, clinical and semantic features for predicting the pathological classification of TETs.

## Materials and Methods

### Patients

**Figure 1:**
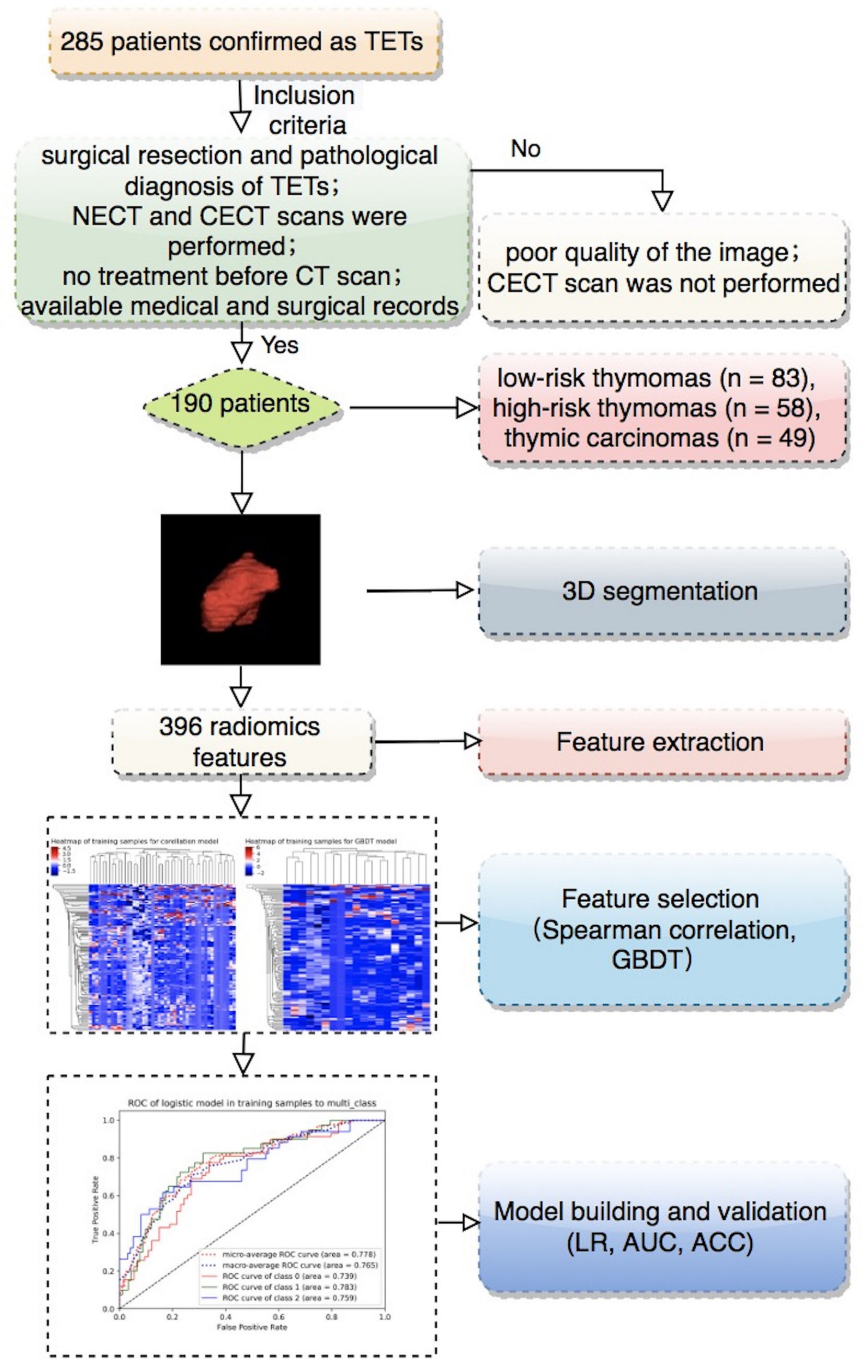
The workflow of this study.

This study was approved by the local ethics committee of our hospital, and the requirement for informed consent was waived. A total of 285 patients who underwent surgical resection and were pathologically confirmed as having TETs in our hospital between July 2012 and December 2019 were retrospectively analyzed. The inclusion criteria were as follows: (1) surgical resection and pathological diagnosis of TET; (2) CECT scan performed within 1 month before surgery; (3) no treatment before CT scan; and (4) available medical and surgical records. The exclusion criteria were as follows: (1) poor image quality due to artefacts or other causes and (2) absence of a CECT scan. Finally, a total of 190 patients were included in our study, and 95 patients were excluded (**Figure 1**). The WHO histologic classification of TETs was determined by surgical conditions and pathological examinations. The clinical features assessed included the following: age, sex, and symptoms (absence of symptoms, chest pain, cough or dyspnoea, myasthenia gravis, weakness, and others).

### Image Acquisition

All patients underwent chest NECT and CECT scans. The scanning equipments were Philips 256 slice iCT of Holland and GE Lightspeed VCT 64 layers of USA. The scanning parameters were as follows: (1) Philips iCT: tube voltage 120 kV, automatic tube current, layer thickness 5 mm, pitch 0.980, reconstruction layer thickness 1 mm; (2) GE Lightspeed VCT: tube voltage 120 kV, tube current 150 mA, slice thickness 5 mm, pitch 0.516, reconstruction layer thickness 0.625 mm. All patients were examined in a supine position, arms up, deep inspiration and scanning. The contrast medium was injected rapidly through the forearm vein using a high-pressure syringe. The contrast agents included iopromide and iohexol. The enhancement phase was delayed for 60 s. The CT images were reconstructed with a standard kernel.

### Tumor Segmentation

**Figure 2:**
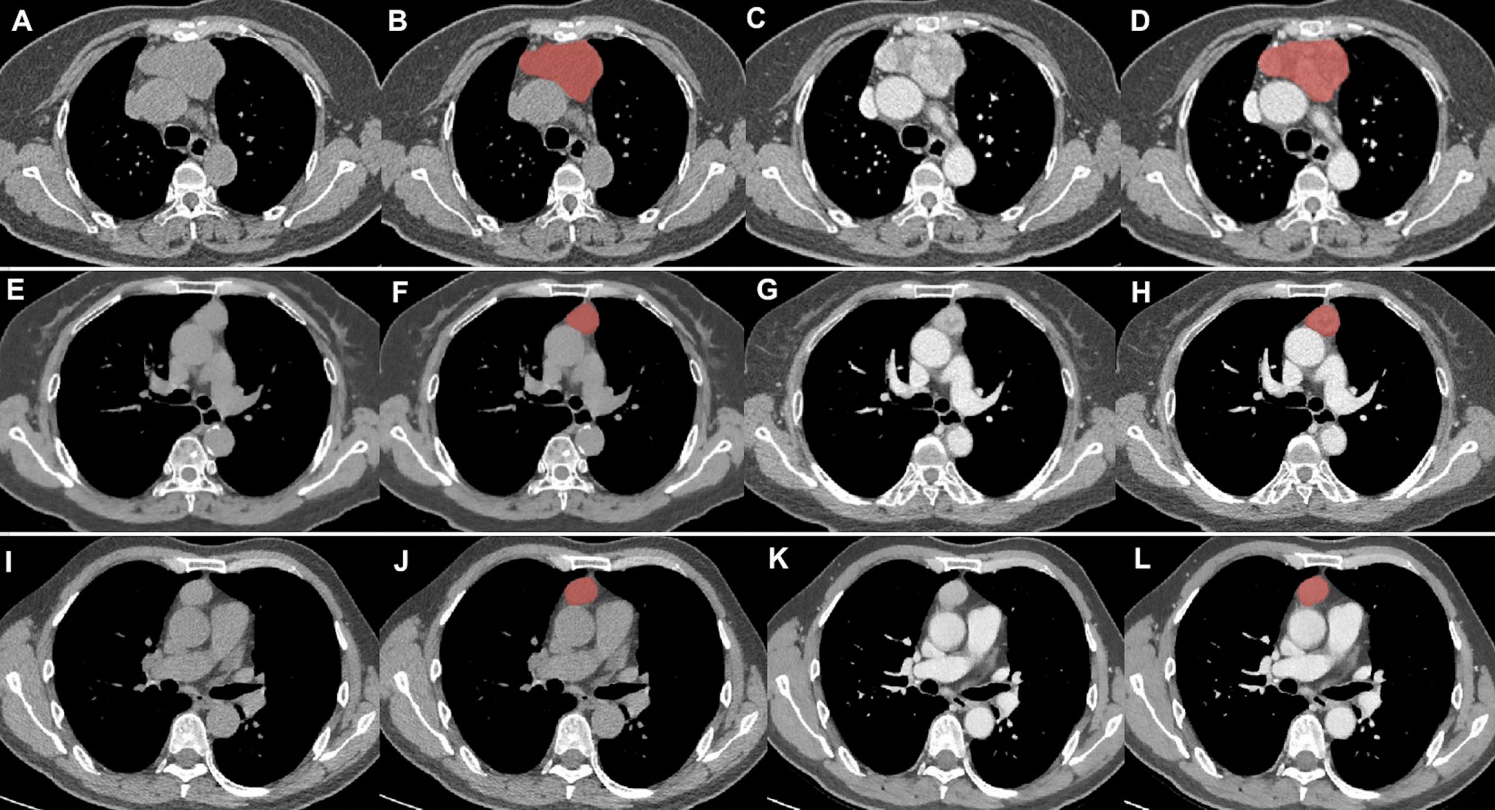
Tumor segmentation. **(A–D)** thymic carcinomas; **(E–H)** high-risk thymomas; **(I–L)**, low-risk thymomas. Columns 1 and 2 were the NECT scan, columns 3 and 4 were the CECT scan. Columns 2 and 4 showed the delineation of different lesions (red areas).

All NECT and CECT Digital Imaging and Communications in Medicine images were exported from the picture archiving and communication system (PACS) of our hospital. ITK-SNAP software version 3.6.0 (www.itksnap.org) was used for manual segmentation. All regions of interest were handcrafted on NECT and CECT images on each slice by a thoracic radiologist with 10 years of experience and validated by a senior thoracic radiologist with 20 years of experience, as shown in **Figure 2**.

### Feature Extraction and Selection

For each patient, a total of 396 radiomics features, including 42 first-order histogram features, 334 second-order texture features, 9 morphological features, and 11 gray-level size zone matrix features, were extracted from all the NECT and CECT images based on Artificial Intelligence Kit software version 3.3.0 (GE Healthcare, China).

We preprocessed the data and normalized the extracted features. When the data value exceeded the range of the mean value and standard deviation, the median of a specific variance vector was used to replace the outliers. In addition, the data were standardized in specific intervals.

In terms of the feature selection method, the Spearman correlation coefficients with a threshold of 0.7 was first used to exclude redundant features. Then, the gradient boosting decision tree (GBDT) algorithm, which has good generalization ability, was used to optimize the subset of features. After the number of features was determined, the most predictive radiomics features were used to construct the final model.

The consistency of features from different machines was evaluated by using intra- and interclass correlation coefficients (ICC). An ICC greater than 0.75 was considered as a good agreement.

### Semantic Features

All images were reviewed by two radiologists, each of whom had more than 10 years of experience in chest CT study interpretation. The two radiologists were blinded to the histologic classification and clinical information. If there was variation in the results, they reviewed the CT images together, and any discrepancies were resolved by discussion until consensus was reached. The semantic features assessed included the following: the maximum diameter of the tumor (measured as the largest cross-section of the mass), cystic degeneration, calcification, mediastinal fat infiltration, mediastinal lymph node enlargement (short diameter >1 cm), and pleural effusion.

### Model Construction and Validation

First, we constructed two triple-classification radiomics classifiers, namely, NECT- and CECT-based RMs, using a logistic regression model. Subsequently, the variables of the clinical features with a *P* value <0.05 were added to the NECT- and CECT-based clinical RMs. Finally, the variables of semantic features with a *P* value <0.05 were added to the NECT- and CECT-based clinical-semantic RMs.

All patients were randomly divided into the training and validation sets at a ratio of 7:3. All three models were trained on the training set by using the repeated five-fold cross-validation method, and the estimation performance of the models was evaluated with the validation set. The performance of different models was assessed using the area under the receiver operating characteristic curve (AUC) and accuracy (ACC).

### Statistical Analysis

All statistical analyses were performed with R (version 3.5.1) and Python (version 3.5.6). Kruskal–Wallis H test was performed to compare continuous variables, whereas the chi-squared test or Fisher’s exact was used for categorical variables amongst groups. All statistical tests were two-sided, and Bonferroni-corrected *P* value was used to identify the feature significance of multiple comparisons.

## Results

### Patient Characteristics

A total of 190 patients (108 males, 82 females; mean age of 51.86 ± 13.09 years, range 24–83 years) were included in this study. Of the patients, 165 underwent thoracoscopic surgery (80 low-risk thymomas, 51 high-risk thymomas, 32 thymic carcinomas) and 25 underwent thoracotomy (3 low-risk thymomas, 7 high-risk thymomas, 17 thymic carcinomas), with a significant difference among the groups (*P* < 0.001). The histopathological results indicated that 83 patients (16 type A, 49 type AB, and 18 type B1) had low-risk thymoma (43 males, 40 females; mean age of 51.25 ± 12.29 years, range 24–79 years), 58 patients (38 type B2 and 20 type B3) had high-risk thymoma (33 males, 25 females; mean age of 47.98 ± 14.04 years, range 24–81 years), and 49 patients (49 type C) had thymic carcinoma (31 males, 18 females; mean age of 57.47 ± 11.46 years, range 31–83 years). The age was significantly different among the groups (*Z* = 7.637, *P* < 0.01). The average age of patients with thymic carcinoma was higher than that of patients in the other two groups. In this study, 101 patients presented no symptoms at tumor diagnosis. For symptomatic patients, the most common symptom in 38 patients was chest discomfort or pain, followed by cough or dyspnea in 18 patients, myasthenia gravis in 14 patients, weakness in 14 patients, and other symptoms in 5 patients. No significant differences were found in sex or symptoms among the three groups (*P* > 0.05; **Table 1**).

**Table 1:** Clinical characteristic of patients.

Variable	Training set					Validation set				
	Low-risk thymoma	High-risk thymoma	Thymic carcinoma	Statistics	*P* value	Low-risk thymoma	High-risk thymoma	Thymic carcinoma	Statistics	*P* value
N	58	40	34			25	18	15		
Age	50.40 ± 11.70	49.85 ± 14.23	57.79 ± 10.76	4.87	0.009	53.24 ± 13.61	43.83 ± 13.04	56.73 ± 13.29	4.316	0.018
Female	28 (48.28%)	17 (42.50%)	14 (41.18%)	0.549	0.76	11 (44.00%)	8 (44.44%)	4 (26.67%)	1.427	0.49
Male	30 (51.72%)	23 (57.50%)	20 (58.82%)			14 (56.00%)	10 (55.56%)	11 (73.33%)		
Symptom				–	0.054				–	0.508
No symptom	37 (63.79%)	23 (57.50%)	14 (41.18%)			11 (44.00%)	8 (44.44%)	8 (53.33%)		
Chest pain	9 (15.52%)	7 (17.50%)	11 (32.35%)			6 (24.00%)	3 (16.67%)	2 (13.33%)		
Cough/dyspnea	3 (5.17%)	3 (7.50%)	4 (11.76%)			4 (16.00%)	1 (5.56%)	3 (20.00%)		
Weakness	6 (10.34%)	3 (7.50%)	0 (0.00%)			3 (12.00%)	1 (5.56%)	1 (6.67%)		
Myasthenia gravis	3 (5.17%)	4 (10.00%)	2 (5.88%)			1 (4.00%)	4 (22.22%)	0 (0.00%)		
Others	0 (0.00%)	0 (0.00%)	3 (8.82%)			0 (0.00%)	1 (5.56%)	1 (6.67%)		

### Semantic Features

The reproducibility of the radiomics features by the different machines was satisfactory (ICC, ranged from 0.771 to 0.905).

In total, six CT image descriptors were developed to characterize the TETs as follows: 1) the mean ± standard deviation of the maximum diameter of the tumor: low-risk thymoma (5.186 ± 2.662 cm), high-risk thymoma (4.857 ± 2.273 cm), thymic carcinoma (5.335 ± 1.914 cm); 2) cystic degeneration (18 low-risk thymomas, 6 high-risk thymomas, 5 thymic carcinomas); 3) calcification (11 low-risk thymomas, 14 high-risk thymomas, 6 thymic carcinomas); 4) mediastinal fat infiltration (7 low-risk thymomas, 13 high-risk thymomas, 30 thymic carcinomas); 5) mediastinal lymph node enlargement (1 low-risk thymoma, 5 high-risk thymomas, 18 thymic carcinomas); and 6) pleural effusion (2 low-risk thymomas, 11 high-risk thymomas, 5 thymic carcinomas). Mediastinal fat infiltration, mediastinal lymph node enlargement, and pleural effusion were significantly different among the groups (*P* < 0.001).

### Radiologic Diagnosis

The radiologists diagnosed 91 low-risk thymomas, 63 high-risk thymomas, and 36 thymic carcinomas. The ACCs of the radiologists’ diagnoses are shown in **Tables 2** and **3**.

**Table 2:** Performance of three-class models in training set.

	AUC	ACC	Precision	Recall	F1-score
NECT-based RM					
Low-risk thymoma	0.739	0.644	0.618	0.810	0.701
High-risk thymoma	0.783	0.644	0.639	0.575	0.605
Thymic carcinoma	0.759	0.644	0.750	0.441	0.556
CECT-based RM					
Low-risk thymoma	0.679	0.576	0.566	0.810	0.667
High-risk thymoma	0.688	0.576	0.536	0.375	0.441
Thymic carcinoma	0.721	0.576	0.667	0.412	0.509
NECT-based clinical-RM					
Low-risk thymoma	0.746	0.659	0.647	0.759	0.698
High-risk thymoma	0.808	0.659	0.676	0.625	0.649
Thymic carcinoma	0.813	0.659	0.667	0.529	0.590
CECT-based clinical-RM					
Low-risk thymoma	0.690	0.553	0.569	0.707	0.631
High-risk thymoma	0.717	0.553	0.500	0.375	0.429
Thymic carcinoma	0.768	0.553	0.567	0.500	0.531
NECT-based clinical-sematic-RM					
Low-risk thymoma	0.880	0.750	0.731	0.845	0.784
High-risk thymoma	0.850	0.750	0.706	0.600	0.649
Thymic carcinoma	0.939	0.750	0.839	0.765	0.800
CECT-based clinical-sematic-RM					
Low-risk thymoma	0.835	0.705	0.672	0.776	0.720
High-risk thymoma	0.813	0.705	0.731	0.475	0.576
Thymic carcinoma	0.946	0.705	0.744	0.853	0.795
Radiologist diagnosis					
Low-risk thymoma	**-**	0.455	0.551	0.655	0.599
High-risk thymoma	**-**	0.455	0.225	0.225	0.225
Thymic carcinoma	**-**	0.455	0.565	0.382	0.456

AUC, area under curve; ACC, accuracy.

**Table 3:** Performance of three-class models in validation set.

	AUC	ACC	Precision	Recall	F1-score
NECT-based RM					
Low-risk thymoma	0.686	0.483	0.571	0.640	0.604
High-risk thymoma	0.601	0.483	0.350	0.389	0.368
Thymic carcinoma	0.632	0.483	0.500	0.333	0.400
CECT-based RM					
Low-risk thymoma	0.611	0.448	0.514	0.720	0.600
High-risk thymoma	0.574	0.448	0.222	0.111	0.148
Thymic carcinoma	0.626	0.448	0.429	0.400	0.414
NECT-based clinical-RM					
Low-risk thymoma	0.687	0.483	0.500	0.560	0.528
High-risk thymoma	0.699	0.483	0.500	0.444	0.471
Thymic carcinoma	0.689	0.483	0.429	0.400	0.414
CECT-based clinical-RM					
Low-risk thymoma	0.538	0.448	0.486	0.680	0.567
High-risk thymoma	0.644	0.448	0.143	0.056	0.080
Thymic carcinoma	0.679	0.448	0.500	0.533	0.516
NECT-based clinical-sematic-RM					
Low-risk thymoma	0.770	0.569	0.636	0.840	0.724
High-risk thymoma	0.689	0.569	0.500	0.333	0.400
Thymic carcinoma	0.783	0.569	0.462	0.400	0.429
CECT-based clinical-sematic-RM					
Low-risk thymoma	0.785	0.483	0.640	0.640	0.640
High-risk thymoma	0.576	0.483	0.250	0.111	0.154
Thymic carcinoma	0.774	0.483	0.400	0.667	0.500
Radiologist diagnosis					
Low-risk thymoma	**-**	0.448	0.636	0.583	0.609
High-risk thymoma	**-**	0.448	0.261	0.316	0.286
Thymic carcinoma	**-**	0.448	0.462	0.400	0.429

AUC, area under curve; ACC, accuracy.

### Performance of the Different Models

No significant differences were observed in clinical or semantic features between the training and validation groups (*P* > 0.05). After feature selection, 19 NECT features and 19 CECT features remained to construct the RMs (see **Supplementary Material**).

**Figure 3:**
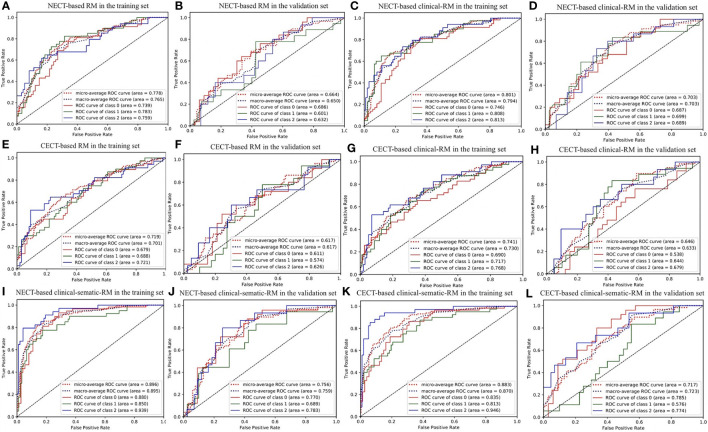
The ROC curve of three-class models. **(A, B)** NECT-based RM in the training and validation sets; **(C, D)** NECT-based clinical-RM in the training and validation sets; **(E, F)** CECT-based RM in the training and validation sets; **(G, H)** CECT-based clinical-RM in the training and validation sets; **(I, J)** NECT-based clinical-sematic-RM in the training and validation sets; **(K, L)** CECT-based clinical-sematic-RM in the training and validation sets. Class 0, low-risk thymoma; class 1, high-risk thymoma; class 2, thymic carcinoma.

In the training set, the NECT-based RM achieved AUCs of 0.739 (for low-risk thymoma), 0.783 (for high-risk thymoma), and 0.759 (for thymic carcinoma) and an ACC of 0.644 (**Table 2**, **Figure 3**). In contrast, the CECT-based RM achieved AUCs of 0.679 (for low-risk thymoma), 0.688 (for high-risk thymoma), and 0.721 (for thymic carcinoma) and an ACC of 0.576. In the validation set, similar results were found, where the NECT-based RM achieved AUCs of 0.686 (for low-risk thymoma), 0.601 (for high-risk thymoma), and 0.632 (for thymic carcinoma) and an ACC of 0.483 (**Table 3**). In contrast, the CECT-based RM achieved AUCs of 0.611 (for low-risk thymoma), 0.574 (for high-risk thymoma), and 0.626 (for thymic carcinoma) and an ACC of 0.448.

When combined with the significantly different clinical features, the clinical RMs performed better than the individual RMs. In the training set, the NECT-based clinical RM exhibited AUCs of 0.746 (for low-risk thymoma), 0.808 (for high-risk thymoma), and 0.813 (for thymic carcinoma) and an ACC of 0.659. Moreover, the CECT-based clinical RM exhibited AUCs of 0.690 (for low-risk thymoma), 0.717 (for high-risk thymoma), and 0.768 (for thymic carcinoma) and an ACC of 0.553. Similarly, in the validation set, the NECT-based clinical RM exhibited AUCs of 0.687 (for low-risk thymoma), 0.699 (for high-risk thymoma), and 0.689 (for thymic carcinoma) and an ACC of 0.483. The CECT-based clinical RM exhibited AUCs of 0.538 (for low-risk thymoma), 0.644 (for high-risk thymoma), and 0.679 (for thymic carcinoma) and an ACC of 0.448.

Finally, when the clinical and semantic features with significant differences were combined, the analysis results showed that the clinical-semantic RMs had the best performance among the three models. In the training set, the NECT-based clinical-semantic RM exhibited AUCs of 0.880 (for low-risk thymoma), 0.850 (for high-risk thymoma), and 0.939 (for thymic carcinoma) and an ACC of 0.750. Moreover, the CECT-based clinical-semantic RM exhibited AUCs of 0.835 (for low-risk thymoma), 0.813 (for high-risk thymoma), and 0.946 (for thymic carcinoma) and an ACC of 0.705. Similarly, in the validation set, the NECT-based clinical-semantic RM exhibited AUCs of 0.770 (for low-risk thymoma), 0.689 (for high-risk thymoma), and 0.783 (for thymic carcinoma) and an ACC of 0.569. The CECT-based clinical-semantic RM exhibited AUCs of 0.785 (for low-risk thymoma), 0.576 (for high-risk thymoma), and 0.774 (for thymic carcinoma) and an ACC of 0.483.

## Discussion

In this study, we found that NECT-based and CECT-based three-class RMs performed well in predicting low-risk thymoma, high-risk thymoma, and thymic cancer, although NECT-based RMs performed better. When combined with clinical data (age only), the clinical RMs performed better than the individual RMs. When the clinical and semantic features with significant differences were combined, the analysis results showed that the clinical-semantic RMs had the best performance among the three models.

In this study, we compared age, sex, and symptoms among the patients with low-risk thymoma, high-risk thymoma, and thymic carcinoma. However, we found a significant difference only in age among the groups, with thymic carcinomas tending to occur at an older average age. Males accounted for slightly more TET cases than females, but there was no difference in the sex composition among the three groups, consistent with previous results (2). Myasthenia gravis is the most important clinical symptom of TETs. Approximately one-third of patients have myasthenia gravis (2). In our study, the incidence of myasthenia gravis was low (less than one-tenth), which may be related to the fact that chest CT has gradually become a routine examination. Most patients were identified through physical examination, while some patients had a cough, usually without symptoms related to myasthenia gravis.

Previous studies have attempted to differentiate low-risk thymoma, high-risk thymoma and thymic carcinoma with conventional CT imaging signs. Some scholars (25) found significant differences in tumor size, contour, adjacent mediastinal fat infiltration, invasion of large vessels, etc. amongst low-risk thymoma, high-risk thymoma, and thymic carcinoma. However, there may be some overlap and lack of specificity in these signs, and the diagnosis is highly dependent on the doctor’s experience. In clinical practice, accurately distinguishing among low-risk thymoma, high-risk thymoma, and thymic carcinoma is still difficult (25). In our study, we compared the maximum tumor diameter, cystic degeneration, calcification, mediastinal fat infiltration, mediastinal lymph node enlargement, and pleural effusion among the low-risk thymoma, high-risk thymoma, and thymic carcinoma groups. The results showed that mediastinal fat infiltration, mediastinal lymph node enlargement, and pleural effusion were significantly different among the groups (*P* < 0.001. We also compared the performance of radiologists in classification *via* RMs. The results showed that the accuracy of the radiologists was lower than that of the RMs. Therefore, identifying quantitative imaging parameters for the histological typing of TETs is essential.

Based on the heterogeneity of tumors, radiomics can non-invasively and quantitatively analyze the characteristics of tumors and monitor the occurrence, development and treatment response of tumors to assist physicians in making clinical decisions (26–28). Previously, some scholars have aimed to distinguish the histological types of TETs through radiomics. Yasaka et al. (29) analyzed the CT images of 39 patients with thymomas and used logistic regression analysis to establish an RM, which exhibited high diagnostic ability. However, the small sample size of the study based on 2D texture analysis did not include thymic carcinoma. Wang et al. (22) analyzed the 3D CT images of 199 patients with thymomas and established an RM by logistic regression analysis. Similarly, thymic carcinoma was not included in that study, and the clinical and semantic features of thymic epithelial neoplasms were not included. Xiao et al. (30) developed a radiological nomogram for predicting TET tissue type by combining a RM, conventional MRI imaging signs and clinical features in multivariable logistic regression analysis and drew a good conclusion.

In our study, we established two triple-classification radiomics classifiers based on the 3D NECT images and CECT images of 190 patients with TETs and analyzed three clinical features and six semantic features. The results showed that the clinical RMs performed better than the individual RMs and that the clinical-semantic RMs had the best performance among the models. However, the AUCs of the radiomics signatures in our study were lower than those in a previous study. We believe that these differences were caused mainly by the different radiomics features extracted from 2D or 3D texture analysis, the different classification methods and different inspection methods. Several studies have shown that compared with 2D texture analysis, 3D texture analysis can improve the classification accuracy (19, 31). Our previous study showed that the results of three classifications are lower than those of two classifications (32). Furthermore, we included both NECT and CECT images and found that NECT-based RMs performed better than CECT-based RMs in predicting low-risk thymoma, high-risk thymoma, and thymic cancer, which we considered to be reasonable. The diagnostic ability of the enhanced image was found to be better than that of the non-enhanced image. However, the enhanced image was observed together with the non-enhanced image rather than separately. In the radiomics approach in our study, the non-enhanced image and the enhanced image were analyzed separately. Moreover, the features extracted from the enhanced and non-enhanced images were different. Therefore, it was possible that the performance of the NECT-based RMs might have been better than that of the CECT-based RMs. A similar situation also arose in our previous studies (33). In clinical practice, NECT-based RMs can provide more diagnostic information in patients who are unsuitable for contrast-enhanced examination.

Our study has several limitations. First, the number of patients was not large. We aimed to reduce false discovery by using training and test cohorts. Despite this approach, we also recognize that TET radiomics data of a larger cohort is necessarily beneficial to validate the model in future studies. Second, multimodal data, such as magnetic resonance (MR) data, may be needed to provide additional useful information for the identification of lesions. Third, in clinical practice, an anterior mediastinal mass that is recommended for surgery may not be a TET. We plan to include more types of anterior mediastinal masses, such as lymphomas and genitourinary tumors, in a later study.

Our study shows that NECT-based and CECT-based RMs may provide a non-invasive method to distinguish low-risk thymoma, high-risk thymoma, and thymic carcinoma. As a quantitative method, radiomics signature analysis can provide complementary diagnostic information, facilitate the development of individualized treatment methods for TET patients, and improve prognosis.

## Data Availability Statement

The original contributions presented in the study are included in the article/**Supplementary Material**. Further inquiries can be directed to the corresponding author.

## Ethics Statement

The studies involving human participants were reviewed and approved by the Peking University People’s Hospital Ethics Review Committee and waived the informed consent. Written informed consent from the participants’ legal guardian/next of kin was not required to participate in this study in accordance with the national legislation and the institutional requirements. Written informed consent was not obtained from the individual(s), nor the minor(s)’ legal guardian/next of kin, for the publication of any potentially identifiable images or data included in this article.

## Author Contributions

1. Guarantor of integrity of the entire study: NH. 2. Study concepts: JL and PY. 3. Study design: JL, PY, and NH. 4. Definition of intellectual content: JL and PY. 5. Literature research: JL, PY, TL, and CS. 6. Clinical studies: JL and PY. 7. Experimental studies: JL and PY. 8. Data acquisition: JL, PY, TL, and CS. 9. Data analysis: PY and SW. 10. Statistical analysis: SW and PY. 11. Manuscript preparation: JL and PY. 12. Manuscript editing: JL and PY. 13. Manuscript review: NH. All authors contributed to the article and approved the submitted version.

## Funding

This work was supported by National Key Research and Development Program of China (No. 2017YFC0109003).

## Conflict of Interest

Author SW was employed by the company GE Healthcare.

The remaining authors declare that the research was conducted in the absence of any commercial or financial relationships that could be construed as a potential conflict of interest.
