# Supplementary material for: CT-Based Radiomics Signatures for Predicting the Risk Categorization of Thymic Epithelial Tumors
Source: Front Oncol. 2021 Feb 26;11:628534. doi: 10.3389/fonc.2021.628534 (PMC7953900; doi:10.3389/fonc.2021.628534)
Supplement: Supplementary file 1 [file DataSheet_1.doc]

Supplementary material Features characteristics of radiomics models

| No. NECT CECT | | |  |
| --- | --- | --- | --- |
| 1 | skewness | MinIntensity | |
| 2 | Uniformity | Percentile80 | |
| 3 | Correlation_angle135_offset7 | uniformity | |
| 4 | GLCMEntropy_AllDirection_offset1_SD | RelativeDeviation | |
| 5 | GLCMEntropy_AllDirection_offset4_SD | stdDeviation | |
| 6 | HaralickCorrelation_AllDirection_offset4_SD | GLCMEntropy_AllDirection_offset4_SD | |
| 7 | GreyLevelNonuniformity_AllDirection_offset4_SD | HGLRE_angle90_offset1 | |
| 8 | HGLRE_AllDirection_offset4_SD | LongRunEmphasis_AllDirection_offset4_SD | |
| 9 | HGLRE_AllDirection_offset7_SD | LRHGLE _AllDirection_offset7_SD | |
| 10 | LongRunEmphasis_angle135_offset1 | LRHGLE _angle135_offset1 | |
| 11 | LongRunEmphasis_angle45_offset7 | LRHGLE _angle90_offset7 | |
| 12 | LRHGLE _AllDirection_offset1_SD | LRLGLE_angle0_offset7 | |
| 13 | LRHGLE _angle45_offset1 | ShortRunEmphasis_angle135_offset1 | |
| 14 | ShortRunEmphasis_angle135_offset1 | SRHGLE _AllDirection_offset1_SD | |
| 15 | SRHGLE _AllDirection_offset1_SD | SRHGLE _AllDirection_offset7_SD | |
| 16 | SRHGLE _AllDirection_offset7_SD | Sphericity | |
| 17 | VolumeMM | HighIntensitySmallAreaEmphasis | |
| 18 | HighIntensityEmphasis | SmallAreaEmphasis | |
| 19 | ZonePercentage | ZonePercentage | |

Note: LRHGLE: Long run high grey level emphasis; SRHGLE: Short run high grey level emphasis; HGLRE: High grey level run emphasis; LRLGLE: Long run low grey level emphasis.
